# Assessment of SARS-CoV-2 Immunity in Convalescent Children and Adolescents

**DOI:** 10.3389/fimmu.2021.797919

**Published:** 2021-12-17

**Authors:** Hing Wai Tsang, Gilbert T. Chua, Kelvin K. W. To, Joshua S. C. Wong, Wenwei Tu, Janette S. Y. Kwok, Wilfred H. S. Wong, Xiwei Wang, Yanmei Zhang, Jaime S. Rosa Duque, Godfrey C. F. Chan, Wai Kit Chu, CP Pang, Paul K. H. Tam, Yu Lung Lau, Ian C. K. Wong, WH Leung, Kwok-Yung Yuen, Mike Y. W. Kwan, Patrick Ip

**Affiliations:** ^1^ Department of Paediatrics and Adolescent Medicine, Li Ka Shing Faculty of Medicine, The University of Hong Kong, Hong Kong, Hong Kong SAR, China; ^2^ State Key Laboratory for Emerging Infectious Diseases, Carol Yu Centre for Infection, Department of Microbiology, Li Ka Shing Faculty of Medicine, The University of Hong Kong, Hong Kong, Hong Kong SAR, China; ^3^ Department of Paediatrics and Adolescent Medicine, Prince Margaret Hospital, Hospital Authority, Hong Kong, Hong Kong SAR, China; ^4^ Department of Pathology, Queen Mary Hospital, Hospital Authority, Hong Kong, Hong Kong SAR, China; ^5^ Department of Ophthalmology and Visual Sciences, The Chinese University of Hong Kong, Hong Kong, Hong Kong SAR, China; ^6^ Department of Surgery and Dr. Li Dak Sam Research Centre, Li Ka Shing Faculty of Medicine, The University of Hong Kong, Hong Kong, Hong Kong SAR, China; ^7^ Faculty of Medicine, Macau University of Science and Technology, Macau, Macau SAR, China; ^8^ Department of Pharmacology and Pharmacy, Li Ka Shing Faculty of Medicine, The University of Hong Kong, Hong Kong, Hong Kong SAR, China

**Keywords:** COVID-19, SARS-CoV-2, convalescence, children, adolescents, T cell response, SARS-CoV-2 RBD IgG

## Abstract

Persistence of protective immunity for SARS-CoV-2 is important against reinfection. Knowledge on SARS-CoV-2 immunity in pediatric patients is currently lacking. We opted to assess the SARS-CoV-2 adaptive immunity in recovered children and adolescents, addressing the pediatrics specific immunity towards COVID-19. Two independent assays were performed to investigate humoral and cellular immunological memory in pediatric convalescent COVID-19 patients. Specifically, RBD IgG, CD4+, and CD8+ T cell responses were identified and quantified in recovered children and adolescents. SARS-CoV-2-specific RBD IgG detected in recovered patients had a half-life of 121.6 days and estimated duration of 7.9 months compared with baseline levels in controls. The specific T cell response was shown to be independent of days after diagnosis. Both CD4+ and CD8+ T cells showed robust responses not only to spike (S) peptides (a main target of vaccine platforms) but were also similarly activated when stimulated by membrane (M) and nuclear (N) peptides. Importantly, we found the differences in the adaptive responses were correlated with the age of the recovered patients. The CD4+ T cell response to SARS-CoV-2 S peptide in children aged <12 years correlated with higher SARS-CoV-2 RBD IgG levels, suggesting the importance of a T cell-dependent humoral response in younger children under 12 years. Both cellular and humoral immunity against SARS-CoV-2 infections can be induced in pediatric patients. Our important findings provide fundamental knowledge on the immune memory responses to SARS-CoV-2 in recovered pediatric patients.

## Introduction

At the end of 2019, a pneumonia outbreak with unknown etiology was reported in Wuhan, China (1, 2). The World Health Organization (WHO) officially named this disease Coronavirus Disease-2019 (COVID-19), which was later identified to be caused by the severe acute respiratory syndrome coronavirus 2 (SARS-CoV-2) (3). The worldwide pandemic has significantly impacted public health and the global economy (4). Preventive measures were enforced to increase social distancing, including limited gatherings, school closures, and restricted travel to reduce transmission (3, 5).

The clinical spectrum of COVID-19 ranges from asymptomatic to fatal disease. Unfavorable outcomes were associated with the age and comorbidities of patients (6, 7), particularly those older than 65 years and individuals with diabetes mellitus or renal disease (8–10). Children infected with SARS-CoV-2 generally have mild symptoms and a low mortality rate (11–13), with a lower likelihood of severe symptoms in children than in adults (14–16). The SARS-CoV-2 viral-host response plays an important role in the pathogenesis of the disease, including changes in the biological responses of peripheral immune cells and the levels of proinflammatory cytokines. Lymphopenia is a common clinical characteristic symptom observed in COVID-19 patients, especially in critical cases (2, 15–20), with up to 83.2% of patients showing lymphopenia during admission (21). Moreover, symptomatic children with COVID-19 were found to have higher viral load, lower total lymphocyte count, lower lymphocyte subsets, and elevated interleukin 6 (IL-6), IL-10, tumor necrosis factor-alpha (TNF-α), and interferon-gamma (IFN-γ) levels compared with asymptomatic patients (22, 23). The data collectively suggest that altered immune cell subsets could be a prognostic factor for COVID-19 (24), especially in critical cases (25). There are knowledge gaps in degree of host immune responses among patients in terms of age, which could help to identify beneficial factors associated with lower disease severity due to SARS-CoV-2 infections.

The long-term persistence of T cell memory is important in mediating both cellular and humoral immunity against SARS-CoV-2 reinfections (26, 27). Patients infected with SARS-CoV-2 virus show T cell memory along with neutralizing antibodies and polyfunctional T cell responses (26, 28). This T cell memory is capable of being reactivated in patients with mild symptoms up to 8 months after recovery (29, 30). Epitope identification studies of SARS-CoV-2 T cells have demonstrated that both CD4+ and CD8+ T cells respond to a broad spectrum of structural and non-structural proteins (NSP) of the SARS-CoV-2 virus. T cells showed immunodominant responses to spike (S), membrane (M), and nuclear (N) structural proteins, whereas B cells showed sub-dominant responses to ORF-1 ab-encoded NSPs (31, 32). However, current knowledge of SARS-CoV-2 immune responses specific to pediatric patients is still lacking, such as the immunodominance of SARS-CoV-2 epitopes and durability of antibodies after an infection.

Given the fundamental differences in the immunity of adults and children (33), we assessed the adaptive SARS-CoV-2-specific immune responses in children and adolescents recovered from COVID-19.

## Materials and Methods

### Subject Recruitment

Children and adolescents under 18 years of age who had recovered from COVID-19 were recruited to the study during the clinical follow up visits. These subjects were admitted and managed in the Paediatric Infectious Disease Centre, Princess Margaret Hospital, Hong Kong, China. Patients were confirmed to have COVID-19 by a positive SARS-CoV-2 RT-PCR test of their nasopharyngeal swab (NPS). Patients were confirmed to have recovered from COVID-19 by either two consecutives negative NPS by SARS-CoV-2 RT-PCR or the seroconversion of SARS-CoV-2 anti-NP antibody response. Details of the admission and discharge criteria and the laboratory investigations have been previously described (5, 23). Briefly, all children and adolescents who were tested positive for SARS-CoV-2 PCR were hospitalized. They were either asymptomatic or had mild diseases (5). Details of the admission and discharge criteria and the laboratory investigations have been previously described (23). Their demographics, clinical symptoms during the infection, and time since recovery were retrieved.

Uninfected controls were recruited from pediatric patients admitted to the Queen Mary Hospital for follow up of other medical conditions unrelated to COVID-19 or from healthy individuals in the community (**Table S1**). Subjects below 18 years of age with no history of COVID-19 and a negative SARS-CoV-2 RT-PCR on the day of recruitment were invited to participate in the study. Exclusion criteria included participants with other acute infections 2 weeks before recruitment, having received any kind of COVID-19 vaccines, known underlying primary or acquired immunodeficiency, and autoimmune disease or other condition that required immunosuppressants.

### Isolation of Peripheral Blood Mononuclear Cells

Whole blood samples from recovered patients and controls were collected in heparin-coated blood tubes. Peripheral blood mononuclear cells (PBMCs) were isolated by Ficoll density gradient centrifugation as previously described (34). Isolated PBMCs were cryo-preserved in storage medium containing 90% heat-inactivated fetal bovine serum (FBS; Gibco, Thermo Fisher Scientific, Inc., Waltham, MA) and 10% cell culture grade DMSO (Sigma Aldrich, Merck, Germany). Samples were stored in liquid nitrogen until batch recovery for the assays.

### T Cell Stimulation Assay and SARS-CoV-2 Peptide Pools

*In vitro* T cell stimulation assays were carried out with spike (S), membrane (M), and nuclear (N) structural proteins. Briefly, viable cell numbers were determined in the thawed PBMCs by staining with crystal violet and counting with a hemocytometer. For the assays, 10^6^ cells were resuspended in 100 μL RPMI 1640 medium (Gibco) supplemented with 10% heat-inactivated FBS and 1% penicillin/streptomycin. The SARS-CoV-2 peptide pools (Miltenyi Biotec, Germany) were prepared according to the manufacturer’s recommendations. Next, 1 μg of peptide/mL (0.6 nmol) separately or in a mixture was introduced to the T cells. Along with the peptide pools, 0.1 μg/mL purified anti-human CD28 (Miltenyi Biotec, Clone: REA612) and 0.1 μg/mL purified anti-human CD49d (Miltenyi Biotec, Clone: MZ18-24A9) as coactivators of T cells were also added to the wells for the entire stimulation period. The T cells and peptide mixtures were incubated at 37°C in 5% CO_2_ for 16 hours. Brefeldin A (Biolegend, San Diego, CA) at a concentration of 0.1 μg/mL was added to the culture medium in the last 4 hours to enhance intracellular cytokine staining signals. The negative control was 10% DMSO and the positive control was an activation cocktail (Biolegend) containing 8.1 nM phorbol-12-myristate (PMA) and 1.3 mM ionomycin.

### Flow Cytometry

Stimulated PBMCs were recovered from the culture plates and resuspended in 100 μL PBS. Cell viability was assessed by staining with Viobility™ Fixable Dyes (Miltenyi Biotec, Germany). Cells were washed, fixed, permeabilized, and then stained with an antibody cocktail containing Pacific Blue™ anti-human CD3 (Biolegend, clone: HIT3a), PE/Cyanine7 anti-human CD4 (Biolegend, clone:A161A1) and PerCP/Cyanine5.5 anti-human CD8 (Biolegend, clone: SK1) for T cell identification; APC anti-human CD69 (Biolegend, clone: FN50) and PE anti-human IFN-γ (Biolegend, clone:4S.B3) for the activation analysis; and FITC anti-human CD14 (Biolegend, clone:HCD14) and FITC anti-human CD20 (Biolegend, clone:2H7) for the exclusion of non-specific signals and B cells. Fifty thousand events were analyzed by a BD LSR-II flow cytometer. (BD Biosciences, San Jose, CA) The gates applied for the quantification of the stimulated T cells are illustrated in **Figure S1**.

### SARS-CoV-2 RBD ELISA

Serum was isolated from whole blood samples obtained from recovered patients and controls. The RBD IgG antibody level was measured using an Euroimmun anti-SARS-CoV-2 ELISA assay (Lubeck, Germany) according to manufacturer’s protocol. Data were expressed as semi-quantitative IgG ratios.

### Quantification and Statistical Analysis

Data analyses were performed using FlowJo (version 10.1, BD Bioscience, Ashland, OR). Statistical analyses were performed using SPSS for Windows (version 26.0, SPSS Inc., Chicago, IL) and Prism for Windows (version 8.0.1, GraphPad Software, San Diego, CA). Data are expressed as mean ± standard deviation (SD), and statistical details are provided in the respective figure legends. Comparison analysis was carried out by two-tailed Student’s t test with p<0.05 considered statistically significant. The antigenicity effect size of the different SARS-CoV-2 peptides on T cell activation was assessed by Cohen’s d (35).

To examine SARS-CoV-2-specific T cell response in recovered patients, we measured the upregulation status of the early activation marker CD69 and expression of intracellular cytokine IFN-γ, a functional T cell marker for protective immunity and analyzed the double-positive status of CD69/IFN-γ in CD4+ and CD8+ T cells, normalized to DMSO control (36–38). To estimate the half-life of SARS-CoV-2 RBD IgG, we calculated t_1/2_ = A_o_/2k, where A_o_ is the initial amount of the antibody obtained from the y-intercept of the trendline and k is the slope of the trendline obtained from the scattered plot of RBD IgG ratio against days after diagnosis. The days after diagnosis is defined as the time between the date of the patient’s clinical diagnosis to the date of the blood sample collections. To analyze the relationship between anti-RBD IgG level and T cells response, we performed Spearman’s correlations and expressed as correlation coefficient (r).

### Ethics Approval

The study was approved by the Institutional Review Board of the University of Hong Kong/Hospital Authority Hong Kong West Cluster (Reference: UW 20-292 and UW 21-157) and the Kowloon West Cluster Research Ethics Committee [Reference: KW/FR-20-086(148-10)]. Written consent was obtained from parents or legal guardians of the subjects.

## Results

### Subject Recruitment and Clinical Characteristics

Between 1^st^ December 2020 to 31^st^ March 2021, 31 patients who had recovered from COVID-19 were recruited from Princess Margret Hospital, Hong Kong SAR. Fourteen (45.2%) were boys and 17 (54.8%) were girls with a median age of 12 years (range 2.7-18 years). The age distribution of the recruited patients was shown in **Figure S2**. Twenty age-matched uninfected controls were also recruited from Queen Mary Hospital, Hong Kong SAR, China and from the community. Subject demographics and clinical characteristics are shown in **Table 1**. The majority of subjects were Chinese (80.6%). Among the COVID-19 cases, 83.9% were domestic cases, 32.3% were asymptomatic, and the remaining cases (67.7%) had mild disease. Blood samples were collected at 29 to 219 days after recovery.

**Table 1:** Demographics and clinical characteristics of recovered pediatric COVID-19 patients and uninfected controls.

	Children Recovered From COVID-19 (N = 31)	Uninfected Controls (N = 20)
Median age in years	12	14
Age range	2.7-18	8-15
Sex (%)		
Male	45.2 (14/31)	80.0 (16/20)
Female	54.8 (17/31)	20.0 (4/20)
Residence (%)		
Hong Kong	100	100
Ethnicity (%)		
Han Chinese	80.6 (25/31)	80.0 (16/20)
Others	19.4 (6/31)	20.0 (4/20)
Travel history (%)		
Yes	16.1 (5/31)	N/A
No	83.9 (26/31)	N/A
Disease awareness (%)		
Asymptomatic	32.3 (10/31)	N/A
Symptomatic	67.7 (21/31)	N/A
Signs/symptoms (%)		
Fever	61.9 (13/21)	N/A
Cough	47.6 (10/21)	N/A
Runny nose	28.6 (6/21)	N/A
Ageusia	19.0 (4/21)	N/A
Vomit	14.3 (3/21)	N/A
Anosmia	9.5 (2/21)	N/A
Sputum	4.9 (1/21)	N/A
Headache	4.9 (1/21)	N/A
SARS-CoV-2 PCR positivity (%)		
Positive	100 (31/31)	N/A
Negative	0 (0/31)	100(20/20)
SARS-CoV-2 anti-NP IgG positivity (%)	100	N/A
Sample collection period	Dec 2020 - March 2021	
Days After Diagnosis	29-219 (Median=46.5)	N/A

N/A, Not Applicable.

### Quantification of SARS-CoV-2 RBD IgG Level and Identification of SARS-CoV-2 Reactive T Cells in Recovered Children and Adolescents

**Figure 1:**
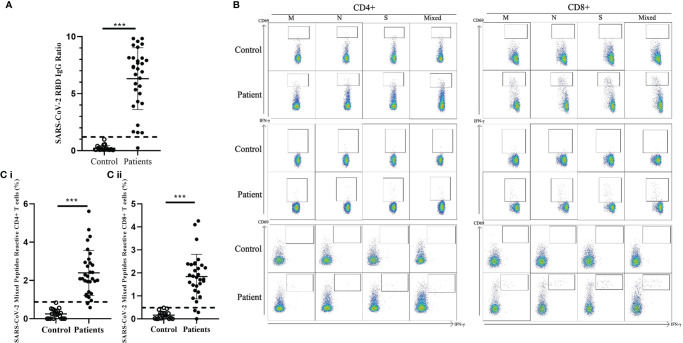
Comparison of SARS-CoV-2 RBD-specific antibodies and SARS-CoV-2-specific T cell response in healthy controls and recovered children and adolescents. **(A)** Serological responses to recombinant RBD protein in 31 recovered COVID-19 patients with median 46.5 recovery days and ranging 29-219 days and 20 uninfected controls. Dash line indicated the anti-RBD IgG ratio reference obtained from uninfected controls. **(B)** Representative data of the T cell response towards SARS-CoV-2 peptide pools in controls and recovered patients. **(C)** (i) CD4+ and (ii) CD8+ T cell responses to SARS-CoV-2 mixed peptides in the recovered COVID-19 patients and uninfected controls. Dash line indicated the measured T cell responses reference obtained from the uninfected controls. Data are presented as mean ± SD and analyzed using two-sided Student’s t-test between control and patient groups. ***p < 0.001.

We detected the presence of SARS-CoV-2 RBD IgG antibodies in 30/31 recovered COVID-19 patients compared with the 20 healthy unexposed cases (p<0.001), with 1 patient showed negative in the RBD IgG antibodies test (**Figure 1A**). Stimulation of CD4+ and CD8+ T cells with the mixed SARS-CoV-2 peptide pool (S + M + N peptides, representing the reactive epitopes of the SARS-CoV-2 virus) showed significantly higher numbers of CD69+, IFN-γ+, and double-positive CD69+/IFN-γ+ T cells in recovered patients compared with controls.(**Figure 1B**) Significantly higher numbers of CD4+ and CD8+ T cells responding to stimulations by mixed M, N and S peptide pools were observed, with the exception of CD8+CD69+IFN-γ+ subsets that showed statistically marginal differences. (**Table 2**) Overall, 29/31 and 28/31 demonstrated CD4+ and CD8+ T-cell response respectively to SARS-CoV-2 mixed-peptide stimulations at a level above those of the controls. (**Figure 1C**)

**Table 2:** Comparison of SARS-CoV-2 specific T cells subsets in controls and recovered children and adolescents.

			M (Mean ± SD)	p-value	N (Mean ± SD)	p-value	S (Mean ± SD)	p-value	Mixed (Mean ± SD)	p-value
CD4	CD69+	Control	0.183 ± 0.177	<0.0001 ***	0.193 ± 0.253	<0.0001 ***	0.238 ± 0.212	<0.0001 ***	0.241 ± 0.224	<0.0001 ***
		Patients	1.039 ± 0.692		0.933 ± 0.573		1.295 ± 0.786		1.957 ± 1.084	
	IFN-γ+	Control	0.036 ± 0.068	<0.0001 ***	0.022 ± 0.038	<0.0001 ***	0.085 ± 0.140	0.0023 **	0.015 ± 0.039	<0.0001 ***
		Patients	0.252 ± 0.191		0.225 ± 0.183		0.232 ± 0.186		0.282 ± 0.217	
	CD69+/IFN-γ+	Control	0.017 ± 0.027	0.0016 **	0.019 ± 0.029	<0.0001 ***	0.030 ± 0.036	0.0001 ***	0.021 ± 0.023	<0.0001 ***
		Patients	0.070 ± 0.080		0.083 ± 0.058		0.108 ± 0.093		0.156 ± 0.136	
CD8	CD69+	Control	0.073 ± 0.085	0.0001 ***	0.118 ± 0.204	0.0009 ***	0.091 ± 0.132	0.0001 ***	0.136 ± 0.182	<0.0001 ***
		Patients	0.392 ± 0.395		0.490 ± 0.523		0.468 ± 0.459		1.302 ± 0.775	
	IFN-γ+	Control	0.046 ± 0.047	<0.0001 ***	0.083 ± 0.152	0.0001 ***	0.073 ± 0.145	0.0004 ***	0.053 ± 0.088	<0.0001 ***
		Patients	0.259 ± 0.186		0.341 ± 0.274		0.275 ± 0.237		0.378 ± 0.280	
	CD69+/IFN-γ+	Control	0.012 ± 0.022	0.0050 **	0.012 ± 0.034	0.0263 *	0.028 ± 0.049	0.0517	0.011 ± 0.020	0.0060 **
		Patients	0.049 ± 0.065		0.097 ± 0.197		0.106 ± 0.209		0.159 ± 0.279	

Immunophenotyping of PBMCs for frequency of CD4+, CD8+, or CD69+ T cells, IFN-γ+ cells, and CD69+/IFN-γ+ double-positive cells from uninfected individuals (n=20) or convalescent children and adolescents (n=31). Data are presented as mean ± SD and analyzed using two-sided Student’s t-test between control and patient groups. *p<0.05, **p<0.01, ***p<0.001

**Figure 2:**
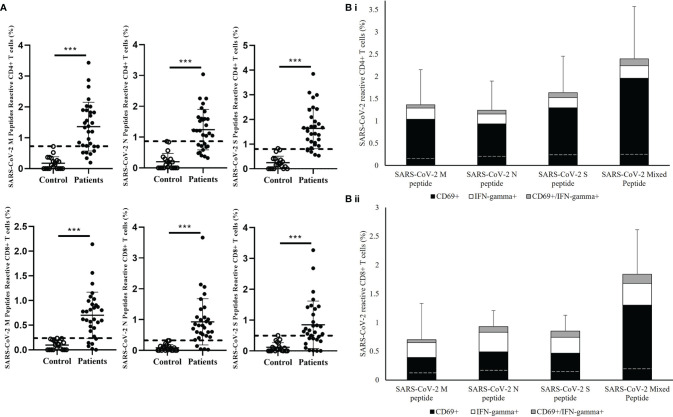
Measurement of SARS-CoV-2-specific T cell response in in healthy controls and recovered pediatrics patients. **(A)** Comparison of T cell responses stimulated by SARS-CoV-2 Membrane (M), Nuclear (N), Spike (S) peptides in the recovered COVID-19 patients and uninfected controls. Dash line indicated the measured T cell responses reference obtained from the uninfected controls. Data are presented as mean ± SD and analyzed using two-sided Student’s t-test between control and patient groups. ***p < 0.001 **(B)** Total T cell responses towards SARS-CoV-2 M, N,S peptides and mixed peptide pools in stacked columns representing the summation of different measured immune subsets in (i) CD4+ and (ii) CD8+ T cells after 16 hours of incubation of PBMCs from recovered patient. Data are expressed as mean ± SD. Dash line in the stack columns indicated the corresponding reference CD4+ and CD8+ T cells response stimulated by different SARS-CoV-2 peptide in uninfected controls group.

Next, the reactivity of the CD4+ and CD8+ T cells towards individual M, N, and S peptide pools were analyzed in convalescent patients. (**Figure 2**) SARS-CoV-2 reactive CD4+ and CD8+ T cells were detectable towards each structural protein in most of the patients’ samples (**Figure 2A**); 24/31 and 25/31 showed stronger CD4+ and CD8+ T cells response respectively to SARS-CoV-2 M peptide stimulation than control. Similar response levels were also observed in CD4+ T cells stimulated by SARS-CoV-2 S peptide and CD8+ T cells stimulated by SARS-CoV-2 N peptide with 27/31 showed higher response than control. However, relatively lower response was observed in both S peptide stimulated CD8+ T cells and N peptide stimulated CD4+ T cells with 11 patients showed similar response to control. Overall summation analysis on the T cells response towards SARS-CoV-2 peptides stimulation was demonstrated. (**Figure 2B**) The CD4+ T cells responded more strongly to stimulation by S peptide than to N (Cohen’s d=0.53) or M peptides (Cohen’s d=0.34). On the other hand, CD8+ T cells responded less strongly to stimulation by M peptides compared with N peptides (Cohen’s d=-0.36) or S peptide (Cohen’s d=-0.23), where the difference in CD8+ T cell responses between S and N peptides was small (Cohen’s d=0.10).

### The Dynamics of Humoral and Cellular Immunity in Recovered Children and Adolescents

**Figure 3:**
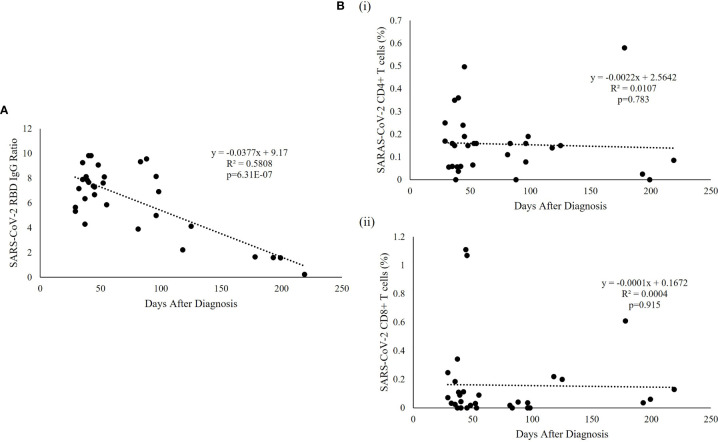
SARS-CoV-2-specific RBD and T cell responses over time. **(A)** Regression analysis of the measured RBD IgG ratio in convalescent serum was plotted against the days after diagnosis. The best fitting trendline is shown. The calculated t_1/2_ was 121.6 days and the estimated duration of antibodies was 7.9 months compared with the average basal level obtained from uninfected individuals. **(B)** Representative T cell subset frequencies in PBMC of recovered patients were plotted against the post-infection period showing a flat slope for (i) CD4+ and (ii) CD8+, indicating a sustained T cell response to SARS-CoV-2 virus in recovered pediatric patients.

SARS-CoV-2 specific humoral immunity was found to decay over time, but not T cell immunity (**Figure 3**). Linear regression analysis showed that the level of SARS-CoV-2 RBD IgG was significantly associated with days after diagnosis (p=6.31^e-07^, R^2^ = 0.5808) (**Figure 3A**), but not with the specific CD4+ (p=0.783) or CD8+ (p=0.915) T cell responses (**Figure 3B**). SARS-CoV-2 RBD IgG had a fast decay rate (-0.0377 anti-RBD IgG ratio/day) while CD4+ (-0.0022%/day) and CD8+ (-0.0001%/day) T cell responses persist over time, including the patient with the longest follow-up time at 219 days who had undetectable anti-RBD IgG but persistent SARS-CoV-2 specific CD4+ and CD8+ T-cell response. The average SARS-CoV-2 RBD IgG half-life (t_1/2_) decay was 121.6 days, and the presence of antibodies was estimated to last for 237.7 days or 7.9 months. The same estimation was not applicable to CD4+ and CD8+ T cell responses because of the lack of association with time.

### Age Is a Factor Associated With the Measured RBD IgG Level and T Cell Activation Magnitudes in Recovered Children and Adolescents

**Figure 4:**
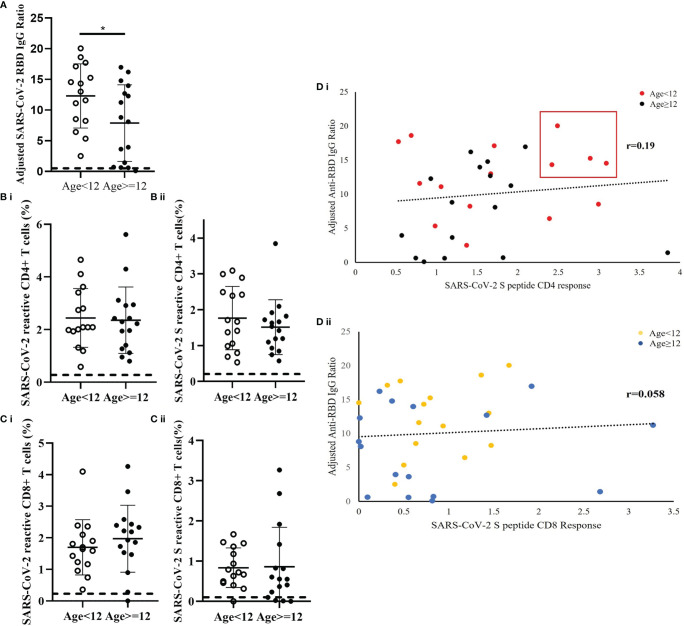
Age-dependent differences of SARS-CoV-2-specific S-RBD IgG level and SARS-CoV-2-specific T cell response in recovered children and adolescents. The corresponding reference anti-RBD IgG ratio and T cell response obtained from uninfected control was indicated as a dash line in the figures. **(A)** Serological analysis in 15 patients who were younger than 12 years and 16 patients who were 12 years or older. Data was adjusted by days after diagnosis and comparisons analyzed by two-sided Student’s t-test *p<0.05. **(B)** Comparison analysis of the total measured CD4+ T cell responses to (i) mixed peptide pools and (ii) S peptide between younger children and older children. **(C)** Comparison analysis of the total measured CD8+ T cell responses to (i) mixed peptide pools and (ii) S peptide between younger children and older children. **(D)** Correlation analysis of anti-RBD IgG level against (i) CD4+ and (ii) CD8+ T cells response in the recovery patients. Data was plotted as age-subgroups with color-labelled dots in the scattered plots. A trendline indicated the correlations direction of the analysis parameters.

Fifteen patients were younger than 12 years and 16 patients were 12 years or older. The results demonstrated differences in the immune responses to SARS-CoV-2 between older and younger children. In comparison to children older than 12 years, the younger patients had a significantly higher level of SARS-CoV-2 RBD IgG ratio (p=0.041) (**Figure 4A**). While the frequency of CD4+ T cells reactive to mixed M, N and S peptide pool was similar between the age groups (Cohen’s d=0.071) [**Figure 4B**(i)], the frequency of S-peptide specific CD4+ T cells was higher in younger children (Cohen’s d=0.3058) [**Figure 4B**(ii)]. Correlative analysis showed that the four patients with highest level of anti-RBD IgG and S-peptide specific CD4+ T cells were all from the younger age group [**Figure 4D**(i)]. In contrast, no difference was observed between the two age groups in SARS-CoV-2 S-reactive CD8+ T cells (Cohen’s d=0.03164) [**Figures 4C, 4D**(ii)].

## Discussion

This study characterizes SARS-CoV-2-specific humoral and cellular immunity in children recovered from COVID-19. There was acquired immunity observed in children with either symptomatic or asymptomatic infections. Both SARS-CoV-2-specific humoral and cellular immunity were detectable at different time points during the recovery period. Detection of SARS-CoV-2 RBD IgG and reactive CD4+ and CD8+ T cells against the various peptide pools suggests broad humoral and cellular immunity are present that can counter re-infections.

Our study showed that there were both CD4+ and CD8+ T cell responses to SARS-CoV-2 S, N, and M proteins. The observed up-regulated production of intracellular IFN-γ in our patients cohort was similar to previous published adults studies, suggesting the protective cellular immunity elicited by the T cell memory towards SARS-CoV-2 was also developed in children and adolescents (26, 30, 39). A larger-scale study will be needed to confirm our observations.

The persistence of humoral and cellular responses against the SARS-CoV-2 virus is key to understanding the risk of re-infections (40, 41). We observed a decline in humoral immunity associated with days after diagnosis. The SARS-CoV-2 RBD IgG antibody level lasted on average 7.9 months with a half-life of 121.6 days, which is similar to other studies across different age groups (42–47). There have only been a few studies demonstrating the longevity of SARS-CoV-2 T cell response in recovered pediatrics patients. Dan et al., demonstrated that approximately 92% and 50% of recovered patients had specific CD4+ and CD8+ responses, respectively, up to 8 months after the primary infection (30). Based on our finding and the above study, the humoral immunity against SARS-CoV-2 in recovered pediatrics patients can last up to 7-8 months after the primary infection, which seems shorter than cellular immunity.

Ding et al., demonstrated an age-specific variation in childhood CD4+ and CD8+ T cell subsets in healthy Chinese, suggesting differences in immune composition across pediatric age groups (48). Along with this finding, our data demonstrated that the age of the pediatric patients is an important factor influencing the level of SARS-CoV-2 RBD IgG and the magnitude of the T cell response to SARS-CoV-2. Recovered children younger than 12 years had higher SARS-CoV-2 RBD IgG levels. There was also age-dependent CD4+ T cell activity in the production of the RBD IgG antibody. Based on our data, we demonstrated an unreported observation of stronger SARS-CoV-2 S CD4+ T cells response correlated with higher level of anti-RBD IgG ratio in younger children. Our novel findings on the immune responses in convalescent pediatrics patients in younger age group underscored the importance of SARS-CoV-2 S specific CD4+ dependent humoral response in relations to the level of anti-RBD IgG against reinfections, which warrant further larger-scale studies to confirm the observations.

The study findings need to be interpreted with the following caveats. First, the number of patients and controls was relatively small. However, all the controls demonstrated negative immune memory responses with undetectable SARS-CoV-2 anti-RBD antibody titer, indicating immune protection against SARS-CoV-2 in unvaccinated and undiagnosed children were minimal. Second, the duration of follow-up was limited and unevenly distributed, it may affect the correlation analysis in determining the kinetics of SARS-CoV-2 anti-RBD decays in this study. Third, only SARS-CoV-2 anti-RBD, which targeted the S1 domain of the Spike protein, was investigated in this study. Other protective neutralizing antibodies targeted to other parts of SARS-CoV-2 spike protein, such as fusion peptide and heptad repeats located in S2 domain, were not evaluated. Last, the quantity of blood that can be obtained from younger children is limited, hence, other subsets of T cell responses to SARS-CoV-2 peptide pools were not evaluated in this study. Future investigations should include other T cell subsets such as regulatory T cells and T follicular helper cells (Tfh) to draw a more comprehensive picture of the T cell response against SARS-CoV-2 in children.

## Conclusion

SARS-CoV-2 infection induces immune memory in recovered pediatrics patients. The T cell reactivity upon stimulation by M, N, S peptide pools in recovered pediatric patients were similar. There were differences in the level of SARS-CoV-2 RBD IgG and the magnitude of T cell responses between younger and older children. Our findings pave the way for large-scale studies, which could help explain the differences in clinical findings between children and adults with COVID-19. Our findings also have important implications for the development of COVID-19 vaccines targeting younger children.

## Data Availability Statement

The raw data supporting the conclusions of this article will be made available by the authors, without undue reservation.

## Ethics Statement

The studies involving human participants were reviewed and approved by Institutional Review Board of the University of Hong Kong/Hospital Authority Hong Kong West Cluster (Reference: UW 20-292 and UW 21-157) and the Kowloon West Cluster Research Ethics Committee [Reference: KW/FR-20-086(148-10)]. Written informed consent to participate in this study was provided by the participants’ legal guardian/next of kin.

## Author Contributions

PI is the Principal Investigator of the Collaborative Research Fund, the funding source of this study. PI, HWT, and GTC contributed to the study conception and research design. MK, KWT, WW, and WHL contributed to the data collection and analysis. PI, HWT, GTC, YLL, WWT, and JK contributed to the data interpretation. HWT, XW, and YZ contributed to the experimental sample preparation and processing. HWT and GTC drafted the manuscript and all co-authors commented and contributed to the revisions and final manuscript.

## Funding

This work was supported by the Hong Kong Collaborative Research Fund (CRF) 2020/21 and the CRF Coronavirus and Novel Infectious Diseases Research Exercises (Reference Number: C7149-20G), and the Health and Medical Research Fund (Reference number: COVID190106). The funding sources were not involved in the study design, data collection, analysis, and interpretation, writing of the manuscripts, and the decision to submit the manuscript for publication.

## Conflict of Interest

The authors declare that the research was conducted in the absence of any commercial or financial relationships that could be construed as a potential conflict of interest.

## Publisher’s Note

All claims expressed in this article are solely those of the authors and do not necessarily represent those of their affiliated organizations, or those of the publisher, the editors and the reviewers. Any product that may be evaluated in this article, or claim that may be made by its manufacturer, is not guaranteed or endorsed by the publisher.

## Supplementary Material

Supplementary Table 1 Other medical conditions in the controls.

Supplementary Figure 1 Representative gating analysis by flow cytometry. **(A)** Forward scatter height (FSC-H) versus forward scatter area (FSC-A) plot for single cell inclusion. **(B)** Live cells were gated based on live/dead discrimination dye staining. **(C)** Side scatter (SSC-A) versus Forward scatter area (FSC-A) plot for lymphocyte identification. **(D)** T cells were gated based on specific CD3 expression, excluding CD14 and CD20 expressing cells. **(E, F)** Subsets of T cells were gated based on the high expression of CD4 and CD8, and were used in further marker analysis. Floating gates on CD69, IFN-γ, and double-positive CD69/IFN-γ plots were based on the corresponding expressions of the positive control cells.

Supplementary Figure 2 Age distribution of the recovered COVID-19 pediatrics patients.
