# Supplementary figures and images for: Assessment of SARS-CoV-2 Immunity in Convalescent Children and Adolescents
Source: Front Immunol. 2021 Dec 17;12:797919. doi: 10.3389/fimmu.2021.797919 (PMC8718543; doi:10.3389/fimmu.2021.797919)

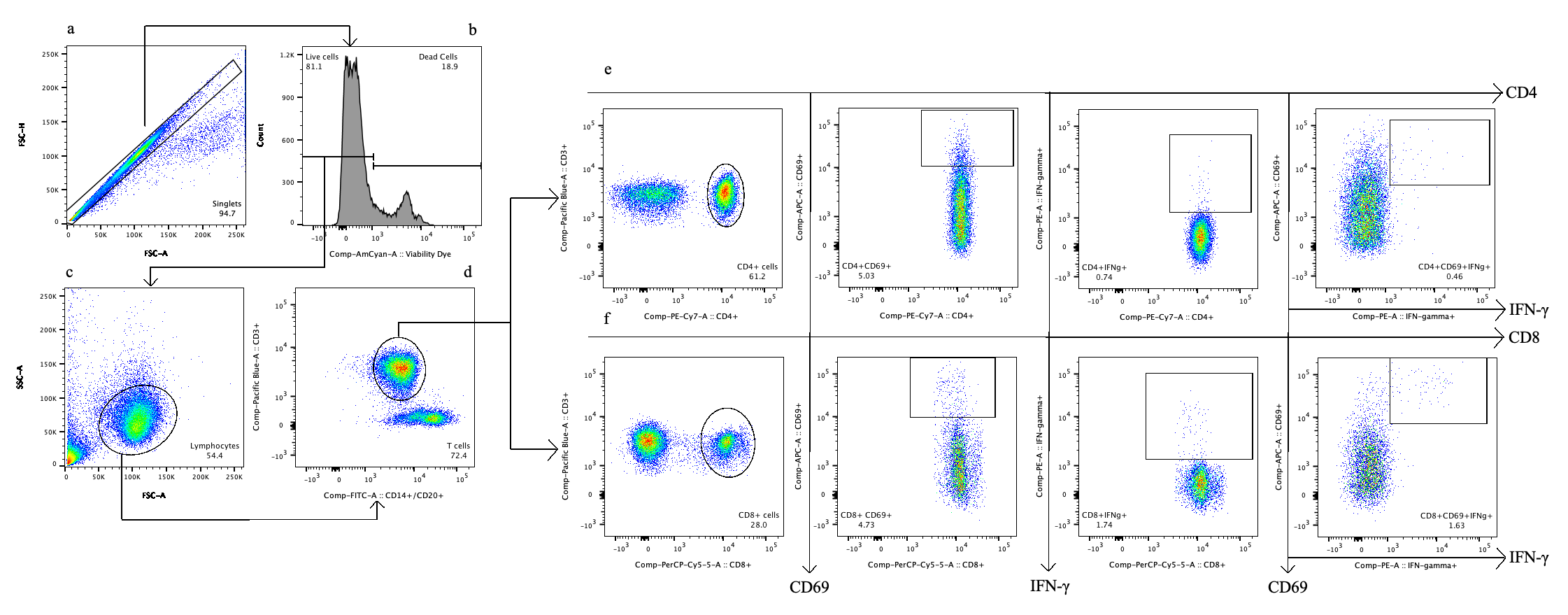

Supplement: Supplementary Figure 1 — Representative gating analysis by flow cytometry. (A) Forward scatter height (FSC-H) versus forward scatter area (FSC-A) plot for single cell inclusion. (B) Live cells were gated based on live/dead discrimination dye staining. (C) Side scatter (SSC-A) versus Forward scatter area (FSC-A) plot for lymphocyte identification. (D) T cells were gated based on specific CD3 expression, excluding CD14 and CD20 expressing cells. (E, F) Subsets of T cells were gated based on the high expression of CD4 and CD8, and were used in further marker analysis. Floating gates on CD69, IFN-γ, and double-positive CD69/IFN-γ plots were based on the corresponding expressions of the positive control cells. [file Image_1.tiff]

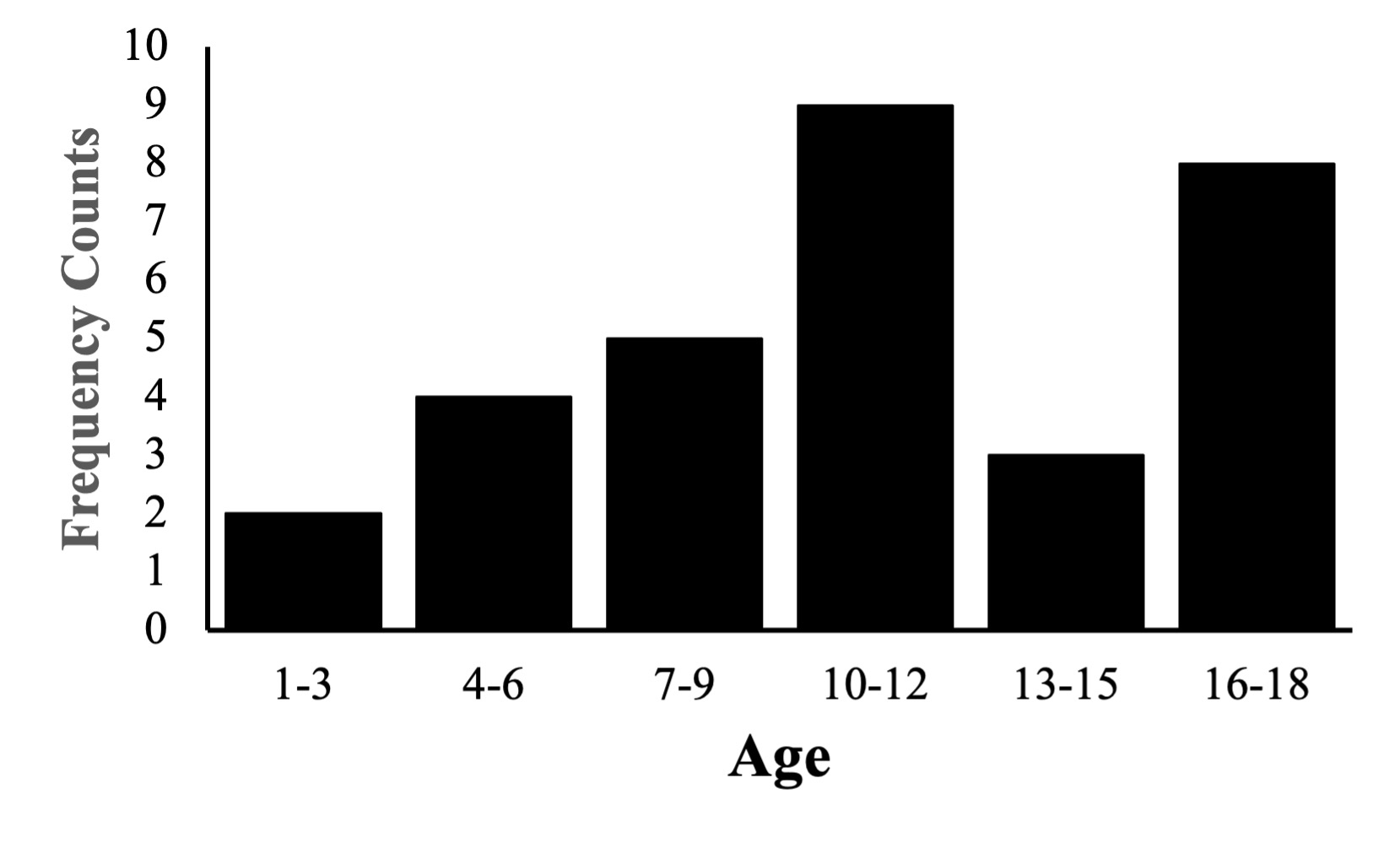

Supplement: Supplementary Figure 2 — Age distribution of the recovered COVID-19 pediatrics patients. [file Image_2.jpeg]
